# Investigating the Production of Foreign Membrane Proteins in Tobacco Chloroplasts: Expression of an Algal Plastid Terminal Oxidase

**DOI:** 10.1371/journal.pone.0041722

**Published:** 2012-07-25

**Authors:** Niaz Ahmad, Franck Michoux, Peter J. Nixon

**Affiliations:** Division of Molecular Biosciences, Imperial College London, London, United Kingdom; University of California – Davis, United States of America

## Abstract

Chloroplast transformation provides an inexpensive, easily scalable production platform for expression of recombinant proteins in plants. However, this technology has been largely limited to the production of soluble proteins. Here we have tested the ability of tobacco chloroplasts to express a membrane protein, namely plastid terminal oxidase 1 from the green alga *Chlamydomonas reinhardtii* (Cr-PTOX1), which is predicted to function as a plastoquinol oxidase. A homoplastomic plant containing a codon-optimised version of the nuclear gene encoding PTOX1, driven by the 16S rRNA promoter and 5′UTR of gene 10 from phage T7, was generated using a particle delivery system. Accumulation of Cr-PTOX1 was shown by immunoblotting and expression in an enzymatically active form was confirmed by using chlorophyll fluorescence to measure changes in the redox state of the plastoquinone pool in leaves. Growth of Cr-PTOX1 expressing plants was, however, more sensitive to high light than WT. Overall our results confirm the feasibility of using plastid transformation as a means of expressing foreign membrane proteins in the chloroplast.

## Introduction

Membrane proteins are involved in an array of biological processes including photosynthesis, respiration, signal transduction, molecular transport and catalysis [1] and constitute around 30% of the proteome [2]. Due to their involvement in cellular communication, they are the targets of more than 50% of current drugs [3] and therefore the focus of drug-discovery programs. However structural studies on membrane proteins lag behind their soluble counterparts: for example, as of December 2011 less than 1% of the more than 50,000 entries in the Protein Data Bank (PDB) repository of protein structures represent membrane proteins, with eukaryotic membrane proteins particularly under-represented [4]. Obtaining sufficient amounts of membrane protein, usually through heterologous expression, is often a major barrier for further detailed structural studies.

Manipulation of the chloroplast genome, mainly in tobacco, has succeeded in producing extraordinary high levels of recombinant proteins such as biopharmaceuticals, vaccine antigens, enzymes and biomaterials, and has provided promising results for engineering agronomic traits in crop plants [5], [6]. Although a large number of recombinant proteins have been expressed in the chloroplast, the potential of the chloroplast to express and process heterologous membrane proteins remains largely unexplored, despite the advantage of having an extensive thylakoid membrane system for targeting proteins [7].

As a first step to test the feasibility of expressing an active foreign membrane protein in the tobacco chloroplast, we chose a predicted plastid/plastoquinol terminal oxidase encoded by the green alga *Chlamydomonas reinhardtii* (Cr-PTOX1) [8]. Successful heterologous expression of *Arabidopsis thaliana* PTOX via the nucleus has been reported in both tobacco [9] and *Arabidopsis* [10] but expression via the plastid genome has not been attempted. PTOX is predicted to be an interfacial membrane protein [11] attached to the stromal-side of the thylakoid laemellae [12], and is involved in oxidation of the plastoquinol pool in the thylakoid membrane [9], with a physiological role in carotenoid biosynthesis [13] and possibly as a safety valve under abiotic stresses such as exposure to high light [14], [15].

We show here that Cr-PTOX1 can indeed be expressed in a functional form in tobacco chloroplasts, and consequently that chloroplasts might be a suitable host for expressing certain types of foreign membrane proteins.

## Results

### Phylogenetic analysis of PTOX1

**Figure 1:**
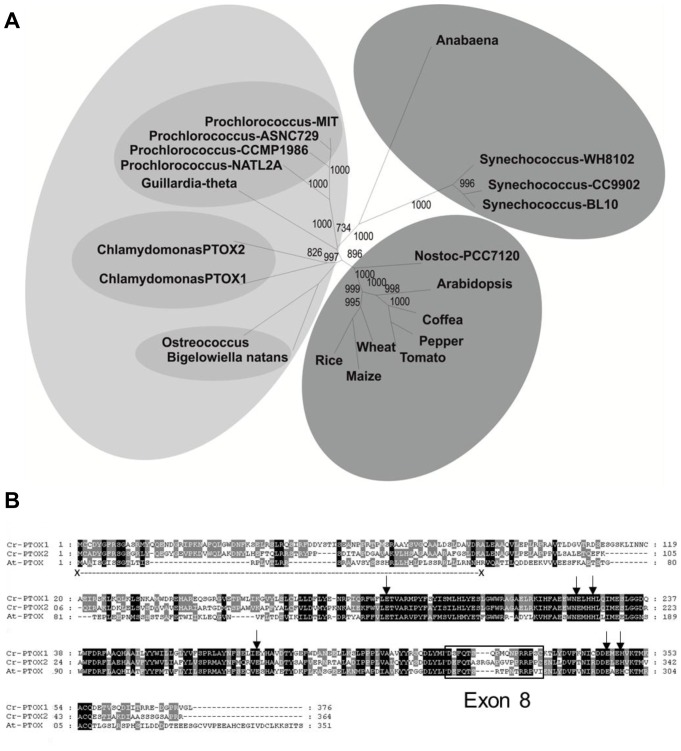
Multiple sequence alignment and phylogenetic analysis of PTOX. (A) Neighbour-joining phylogenetic dendrogram (spider) based upon an alignment of complete amino acid sequences of PTOX molecules. Grouping in different shades based upon structural and functional homology of PTOX polypeptides found in different species. Numbers at nodes indicate bootstrap confidence values (1000 replicates). The PTOX amino acid sequences used were from *Arabidopsis thaliana* GenBank accession number: CAA06190, tomato (*Lycopersicon esculentum*) GenBank accession number: AAG02286, rice (*Oryza sativa*; cultivar *japonica*) NCBI accession number: NP_001054199, wheat (*Triticum aestivum*) GenBank accession number: AAG00450, maize (*Zea mays*) NCBI accession number: NP_001150780, coffee (*Coffea canephora*) GenBank accession number: ABB70513, pepper (*Capsicum annuum*) GenBank accession number: AAG02288, *Guillardia theta* GenBank accession number: CAI77910, *Chlamydomonas reinhardtii* NCBI accession number: AF494290 (PTOX1) and NCBI accession number: XP_001703466 (PTOX2), *Ostreococcus tauri* (green alga) GenBank accession number: CAL58090, *Bigelowiella natans* (chlorarachniophytes) GenBank accession number: AAP79178, *Anabaena variabilis* ATCC 29413 (cyanobacteria) GenBank accession number: ABA21297, Nostoc sp. PCC 7120 (cyanobacteria) NCBI NP_486136, *Prochlorococcus marinus* subsp. Pastoris CCMP1986 (cyanobacteria) NCBI accession number: NP_892455, *Prochlorococcus marinus* Subsp. ASNC729 (cyanobacteria) Genbank accession number: ABE11017, *Prochlorococcus marinus* str. MIT 9312 (cyanobacteria) NCBI accession number: YP_396838, *Prochlorococcus marinus* str. NATL2A (cyanobacteria) NCBI accession number: YP_291624, *Synechococcus* sp. WH 8102 (cyanobacteria) NCBI accession number: NP_896980, *Synechococcus* sp. CC9902 (cyanobacteria) NCBI accession number: YP_376451, and *Synechococcus* sp. BL107 (cyanobacteria) NCBI: ZP_01468216. (B) Multiple sequence alignment of PTOX from *Chlamydomonas* and *Arabidopsis*. Conserved iron-binding residues are indicated by black arrows, whereas, exon 8 is boxed. Conserved sequences are shaded black. The transit peptide for At-PTOX is underlined.

The green alga *Chlamydomonas reinhardtii* encodes two PTOX homologues, PTOX1 and PTOX2 (Figure 1A), with PTOX2 the most important for oxidation of the plastoquinone pool [16]. Although not yet demonstrated experimentally, PTOX1 is also likely to function as a plastoquinol oxidase: it shows 47% sequence identity with *Arabidopsis thaliana* PTOX (IMMUTANS) and has the typical features of a PTOX including the six putative iron-binding sites (E-183, E-222, H-225, E-273, E-324, H-327; numbering according to [17]) [18] and conserved Exon 8 [19], which is required for both the structural as well as functional stability of PTOX [18] (Figure 1B).

### Construction of chloroplast transformation vector for the expression of Cr-PTOX1 in tobacco chloroplasts

Because Cr-PTOX1 was to be expressed within the chloroplast, the sequence encoding the first 41-amino acids, predicted by ChloroP [20] and TargetP [21] to encode the transit peptide, was removed (Figure 1B). In addition a sequence coding for the influenza virus hemaglutinin A tag (HA-tag) was attached to the 5′-end of the *Cr-PTOX1* sequence (GenBank Accession Number AF494290) to allow immunodetection. A custom-made codon-optimized sequence coding for the mature polypeptide was placed under the strong constitutive chloroplast promoter, P*rrn*, in the pHK40 vector [22]. This plasmid targets transgenes into a region of the tobacco plastome between *rrn16-trnV* and *rps12/7* and has been developed to drive high expression of transgenes using a plastid 16S ribosomal RNA gene promoter, and 5′ untranslated region from gene 10 of bacteriophage T7 [22]. The message is stabilized by providing the untranslated 3′ region (T*rbcL*) of the *rbcL* gene encoding the large subunit of ribulose-1,5-bisphosphate carboxylase/oxygenase (Rubisco). An *aadA* cassette provides resistance to spectinomycin.

### Transformation and regeneration of transplastomic plants expressing Cr-PTOX1

**Figure 2:**
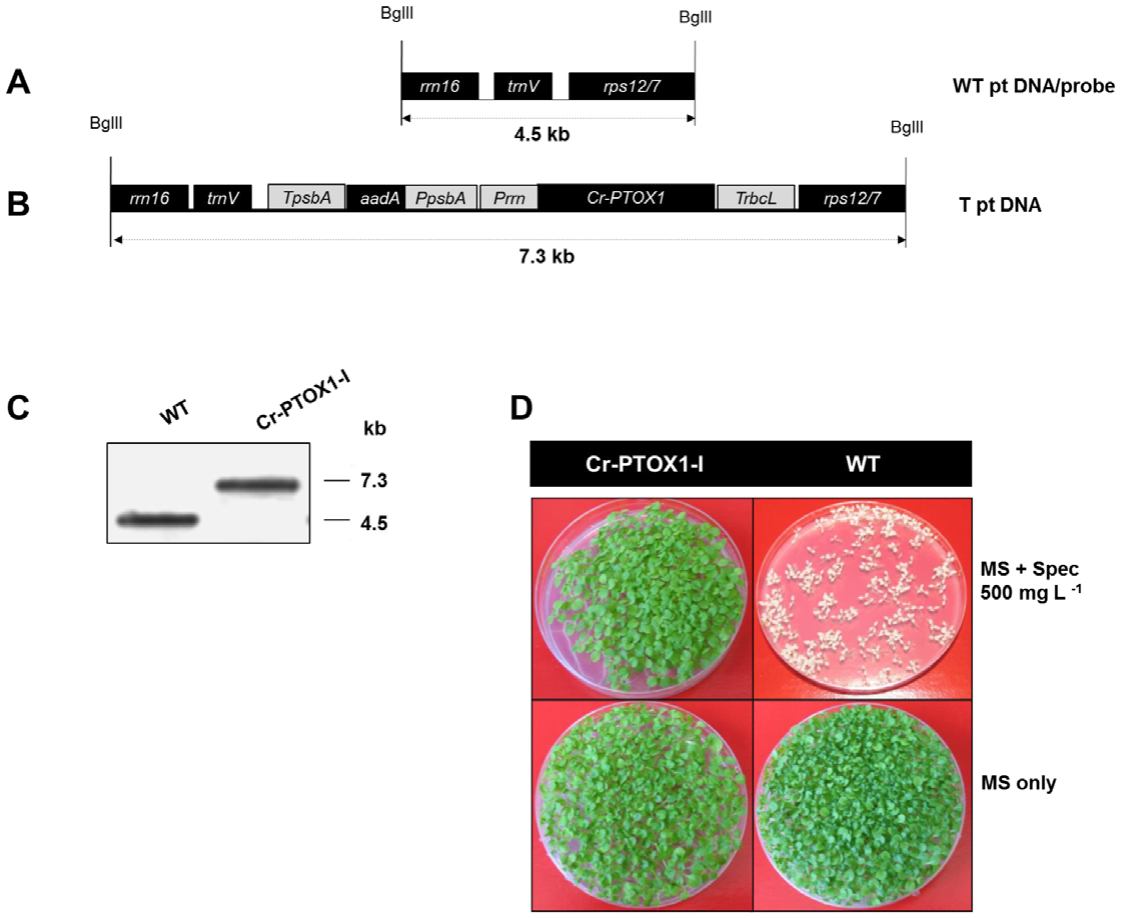
Generation of homoplastomic transplastomic plants expressing Cr-PTOX1. Schematic representation of the plastome region of the wildtype (A) and the transplastomic plant line Cr-PTOX1-I (B) analyzed by Southern blot analysis. Cr-PTOX1-I sequences were cloned in vector pHK40 [22]. The positions of the restriction enzyme BglII used to digest the genomic DNA are shown. The dotted lines represent the size of the expected fragments to be released from wildtype as well as Cr-PTOX1-I after restriction. (C) Total genomic DNA from WT as well as Cr-PTOX1-I was digested and hybridized with rrn16/rps12 digoxigenin labelled probe amplified from WT. (D) Maternal inheritance assay of Cr-PTOX1-I transplastomic plant line. Seeds of Cr-PTOX1-I and WT plants were grown on MS plates with or without 500 mg L^−1^ spectinomycin at room temperature. Abbreviations: P*rrn* = 16SRNA operon promoter, T*rbcL* = Rubisco large subunit terminator, P*psbA* = *psbA* promoter, T*psbA* = *psbA* terminator, WT = wild type, T = transplastomic, ptDNA = Plastid DNA.

The chloroplast shooting vector pNA-PTOX1 was used to insert *Cr-PTOX1* into the tobacco plastome using a particle delivery system [23]. Southern blot analysis confirmed that 1 out of 5 spectinomycin-resistant plants, designated Cr-PTOX1-I, had correctly integrated both *Cr-PTOX1* and the selection cassette at the chosen site in the chloroplast genome, and that the vast majority of plastid DNA copies has been transformed (Figure 2C).

Seeds from Cr-PTOX1-I and WT plants were grown on MS plates with or without 500 mg L^−1^ of spectinomycin to determine the inheritance of the spectinomycin cassette (Figure 2D). Unlike WT, which bleaches in the presence of spectinomycin, all seeds produced by the Cr-PTOX1-I plant line grew normally. The lack of segregation of the selectable marker is a further indication for the incorporation of the transgenes in the chloroplast genome.

### Expression and localization of Cr-PTOX1 in tobacco chloroplasts

RNA gel blot analysis using a *Cr-PTOX1*-specific probe revealed the presence of one major 1.3-kb transcript in Cr-PTOX1-I with no evidence for significant read-through of transcription or accumulation of smaller transcripts (data not shown).

**Figure 3:**
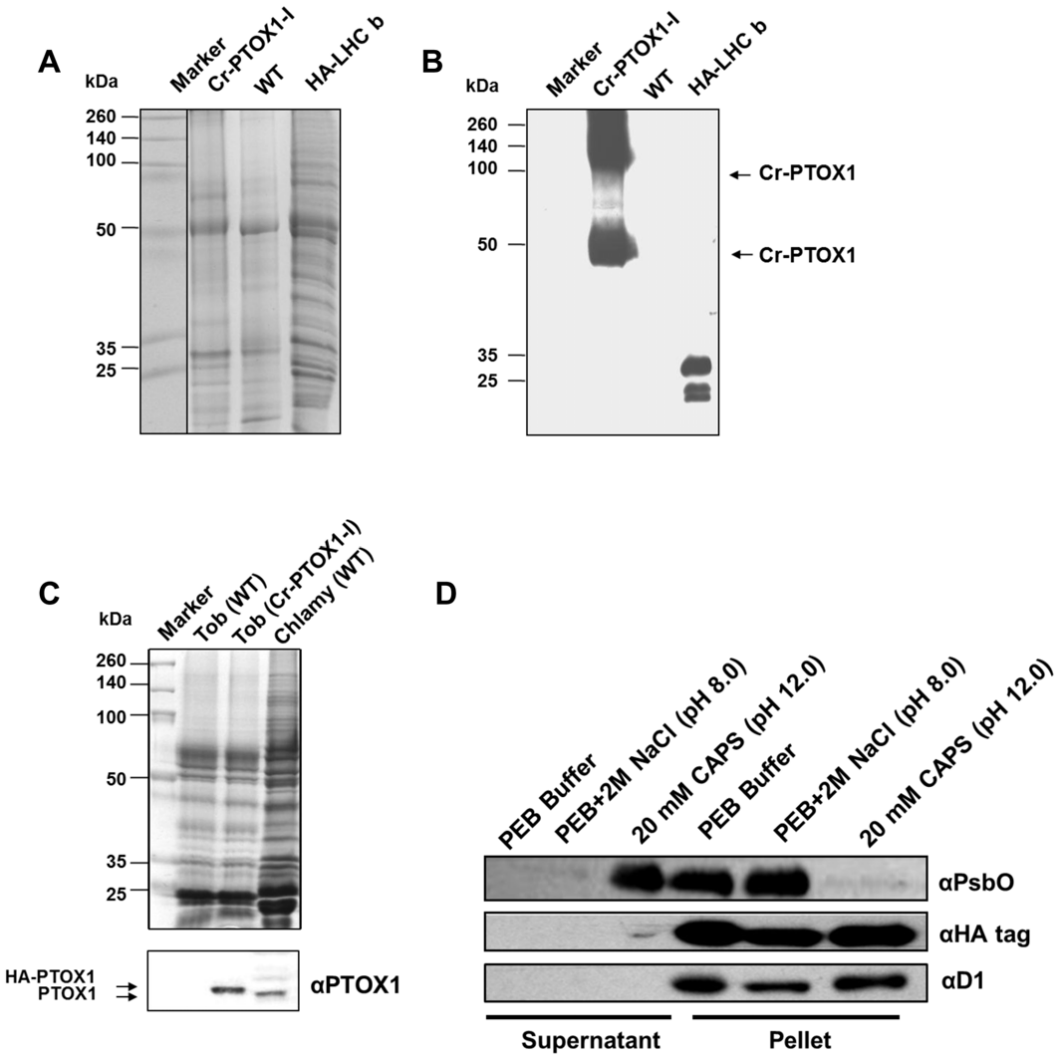
Detection of Cr-PTOX1 by SDS-PAGE and Western blot analysis. Total proteins equivalent to 1 µg of chlorophyll were loaded per well from PTOX1-I plant leaves grown in a greenhouse at high light (125 µmol photons m^−2^ s^−1^) and analysed either by running on a 15% (w/v) denaturing polyacrylamide gel and stained by Coomassie Blue (A) or transferring to PVDF for immunodetection carried out using an anti-HA tag antibody (B). Protein samples from *Chlamydomonas reinhardtii* expressing HA-tagged Light Harvesting Complex b (HA-LHC b) [49] were used as a positive immunoblotting control. (C) Tobacco plants were grown in a growth room at 50 µmol photons m^−2^ s^−1^. 5 µg of thylakoids (based on chlorophyll) from tobacco, wild type (WT) as well Cr-PTOX1 expressing plants and *C. reinhardtii* (wild type) were loaded per well for SDS-PAGE and immunoblotting, which was carried out using an anti-PTOX1 antibody. Upper panel shows a Coomassie-stained gel, whereas, the bottom panel shows the immunoblot. (D) Differential extraction of thylakoids to determine whether Cr-PTOX1 is being targeted to the membrane. Thylakoids extracted from Cr-PTOX1-I plant leaves were washed with different buffers and centrifuged. The supernatant and pellet fractions (∼1 µg chlorophyll per well) were loaded and immunoblotted using different antibodies against membrane bound proteins.

In order to detect the accumulation of Cr-PTOX1 in transplastomic plants, total plant proteins were extracted from Cr-PTOX1-I and WT plants and analysed by SDS-PAGE. No obvious new protein band was detected when the gel was stained with Coomassie Blue (Figure 3A). However, a protein equivalent to the theoretical size of HA-PTOX1 (50.6 kDa) was detected by immunoblotting in Cr-PTOX1-I but not in WT using antibody specific for the HA-tag (Figure 3B). Also, a protein band around twice the size of Cr-PTOX1 protein was consistently detected in all immunoblotting experiments, indicating possible aggregation of Cr-PTOX1.

Further confirmation for the expression of HA-tagged Cr-PTOX1 was obtained in immunoblotting experiments using an anti-peptide antibody specific for Cr-PTOX1 (Figure 3C). As expected HA-tagged PTOX1 migrated more slowly than *bona fide* PTOX1 in *C. reinhardtii* due to the presence of the HA-tag on its N-terminus. A crude comparison of the intensities of the immunoreactive bands suggested that Cr-PTOX1 was expressed in tobacco thylakoids at a higher level than its natural expression in *Chlamydomonas* when normalised to chlorophyll content (Figure 3C).

Membrane washing experiments using buffers of increasing stringency (protein extraction buffer alone (PEB), PEB with 2 M NaCl pH 8.0 and 20 mM CAPS pH 12.0) revealed that Cr-PTOX1, like the integral D1 subunit of photosystem II (PSII), remained membrane-associated under all washing conditions, whereas the more weakly associated extrinsic PsbO protein of PSII was removed by high salt (Figure 3D). These data suggested that Cr-PTOX1 was correctly targeted to the thylakoid membrane and potentially functional.

### Identification of Cr-PTOX1 by native gel electrophoresis

**Figure 4:**
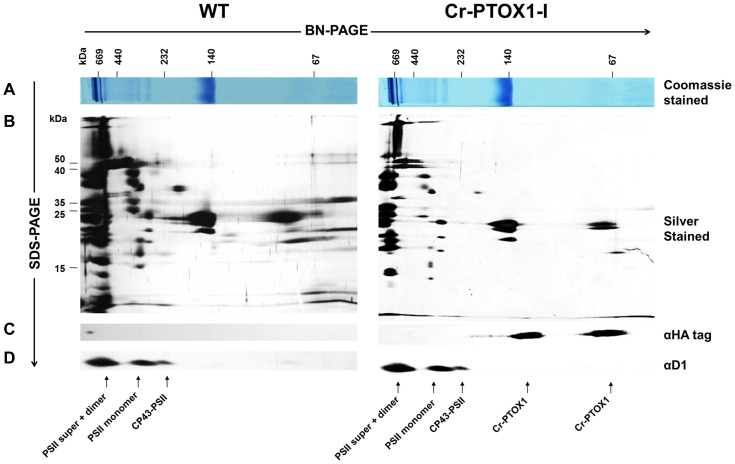
Immunodetection of Cr-PTOX1 protein by BN and 2D SDS-PAGE. Thylakoid membranes from Cr-PTOXI-I or WT containing 4 µg chlorophyll were solubilised and separated by BN-PAGE. One of the BN-PAGE gel lanes was stained with Coomassie blue (A) and others were run in the second dimension by denaturing SDS-PAGE. One of the gels obtained was silver-stained (B) while other two were immunoblotted with anti-HA tag (C) or anti-D1 antibodies (D).

A possible association of Cr-PTOX1 with photosynthetic complexes was assessed by 2D gel electrophoresis using Blue-Native PAGE (BN-PAGE) in the first dimension and denaturing SDS-PAGE in the second (Figure 4). Under the relatively gentle detergent solubilisation conditions used, a range of PSII complexes could be detected using D1-specific antibody including supercomplexes with LHC-II, dimers, monomers and a core complex lacking CP43 (PSII-CP43). Cr-PTOX1, detected using anti-HA-tag antibody, was present in two types of complex: the monomer (about 60 kDa) and a larger, possibly dimeric complex, with a size of 120 kDa (Figure 4C).

### Detection of Cr-PTOX1 activity in transplastomic plants by chlorophyll fluorescence measurements

#### Post-illumination Fo increase in Cr-PTOX1-I plant leaves

Chlorophyll fluorescence measurements were performed to test whether Cr-PTOX1 was expressed in an active state in Cr-PTOX1-I. In the fully oxidized open state, the fluorescence emitted by PSII when probed with weak intensity light is minimal (F_o_) because the energy absorbed by PSII is consumed in electron movement. However, when exposed to saturating light, PSII reaction centres become closed due to reduction of the primary quinone electron acceptor of PSII (Q_A_) and the fluorescence reaches a maximum level (F_m_), which is then quenched through the onset of photochemical and non-photochemical processes associated with CO_2_ fixation and generation of a proton gradient across the thylakoid membrane [24]. Generally, when dark-adapted wild-type plant leaves are illuminated by white light for several minutes and then placed in the dark, a transient increase in ‘apparent’ F_o_ fluorescence takes place because of the reduction of the PQ pool via the NDH complex [25]. This post-illumination F_o_ rise is a well-established method to assay both NDH [26] and PTOX activity [9].

**Figure 5:**
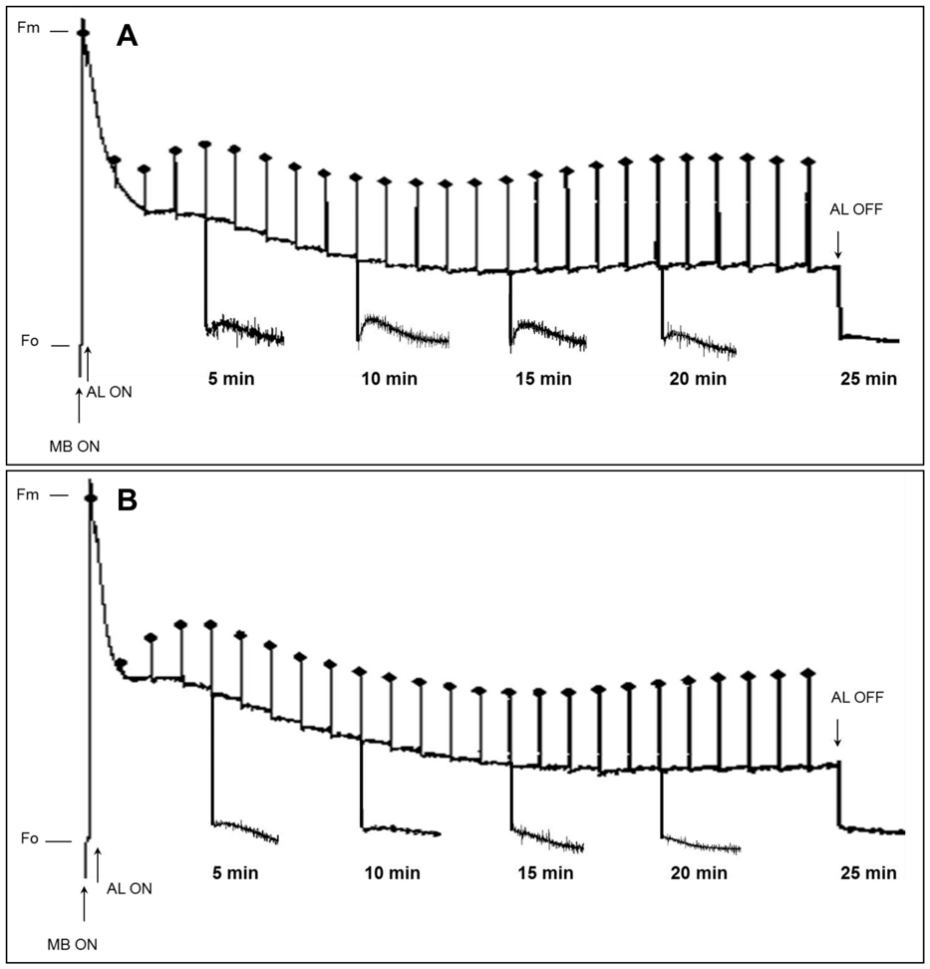
Changes in post-illumination Fo rise at various time points during fluorescence induction. WT and Cr-PTOX1-I plants were grown at low light (50 µmol photons m^−2^ s^−1^). 10-week-old plants were then analysed for post-illumination Fo rise. Dark-adapted WT (A) and Cr-PTOX1-I (B) leaves were illuminated by 96-µmol photons m^−2^ s^−1^ white light (AL) for various time points over a period of 30 min. Post-illumination fluorescence kinetics was monitored by placing leaves in the dark for 5 min after illuminating leaves for 5, 10, 15, 20 and 25 min. Saturating flashes of 1000 µmol photons m^−2^ s^−1^, 600 ms duration each at 1 min interval were applied throughout the period (24 measurements). Abbreviations: AL = Actinic light, MB = Measuring Beam, Fm = Maximum fluorescence from dark-adapted leaves after saturating flash, Fo = minimum fluorescence in dark-adapted leaves before illumination.

The post-illumination F_o_ rise was monitored in both WT and Cr-PTOX1-I plant leaves at various time-points over a 30-min illumination period. In WT, the magnitude of the F_o_ rise was low in the first quarter or so of the illumination period, then increased in the middle and declined at the end (Figure 5A). This post-illumination F_o_ rise was reduced considerably in Cr-PTOX1-I leaves (Figure 5B), consistent with an enhanced oxidation state of the PQ pool in the dark due to enhanced PTOX activity as previously observed with nuclear mutants [9]. The results of this experiment provided strong evidence that *Chlamydomonas* PTOX1 was expressed in an active form in tobacco chloroplasts.

#### Involvement of Cr-PTOX1 in plastoquinol oxidation after flash excitation

**Figure 6:**
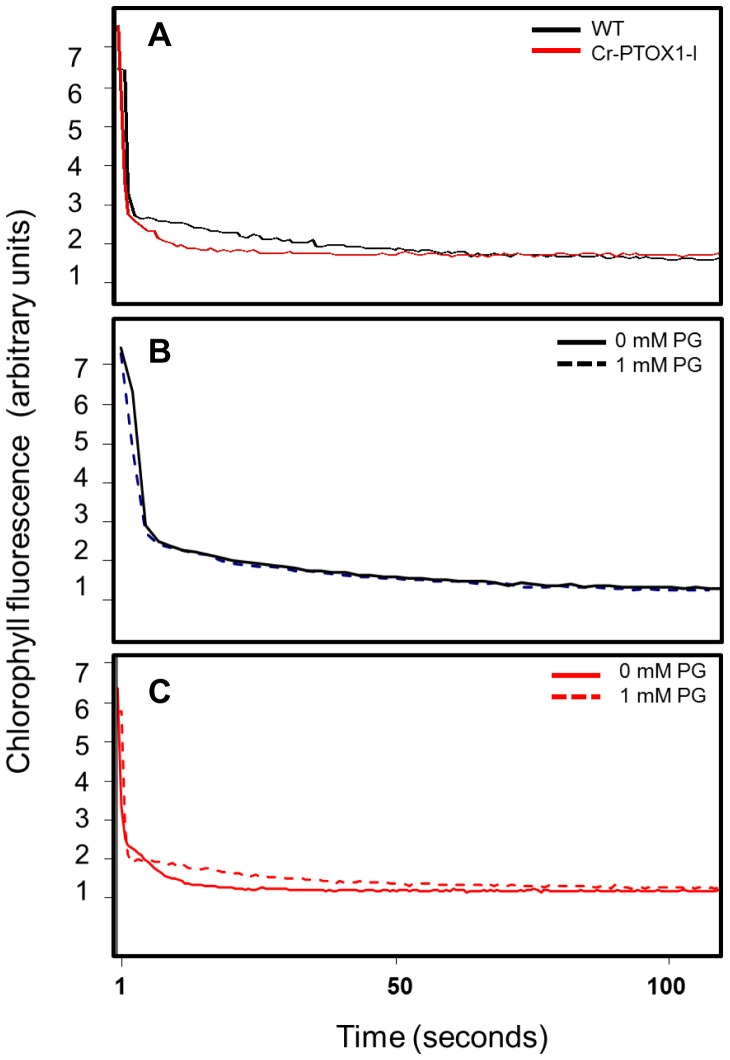
Involvement of Cr-PTOX1 in PQ oxidation and effect of propyl gallate on Cr-PTOX1 activity. WT and Cr-PTOX1-I plants were grown at low light (50 µmol photons m^−2^ s^−1^). 10-week-old plants were then analysed by measuring chlorophyll fluorescence. The fluorescence decay in leaf discs of WT (black trace) and Cr-PTOX1-I plants (red trace) were measured after 1-hour dark adaptation (A). Leaf discs of both WT (B) and Cr-PTOX1-I plants (C) were treated with (broken line) or without (unbroken line) 1 mM propyl gallate (PG) for a period of 3 hours in the dark. Fluorescence values (arbitrary units) are shown on y-axis, whereas, time is shown on x-axis.

Following saturating flash excitation of dark-adapted leaves, the decrease in chlorophyll fluorescence is an indicator of the rate of re-oxidation of the reduced PSII primary acceptor (Q_A_^−^) and consists of both fast and slow phases of oxidation, the latter dependent on the oxidation state of the PQ pool [27]. The fast phase of decay was found to be similar in Cr-PTOX1-I and WT leaves under the measuring conditions used (Figure 6A). However, the slow phase was much faster in Cr-PTOX1-I showing that the re-oxidation of the PQ pool was much quicker in transplastomic Cr-PTOX1-I leaves than in WT (Figure 6A), again in line with previous studies on nuclear transformants [9].

In order to verify that the oxidation of the PQ pool was indeed due to the enhanced activity of PTOX in the chloroplast, Cr-PTOX1-I leaf discs were infiltrated with 1 mM propyl gallate, a known inhibitor of PTOX [8], [9]. No effect of propyl gallate was observed on WT fluorescence (Figure 6B). However, Cr-PTOX1-I now showed a similar slow phase in fluorescence decay to that of the WT (Figure 6C). The results of this experiment are in line with previous observations obtained from over-expression of *Arabidopsis* At-PTOX in tobacco through nuclear transformation [9]. Overall these data support the involvement of Cr-PTOX1 in the oxidation of the PQ pool.

### Transplastomic expression of Cr-PTOX1 promotes sensitivity to high-light stress

**Figure 7:**
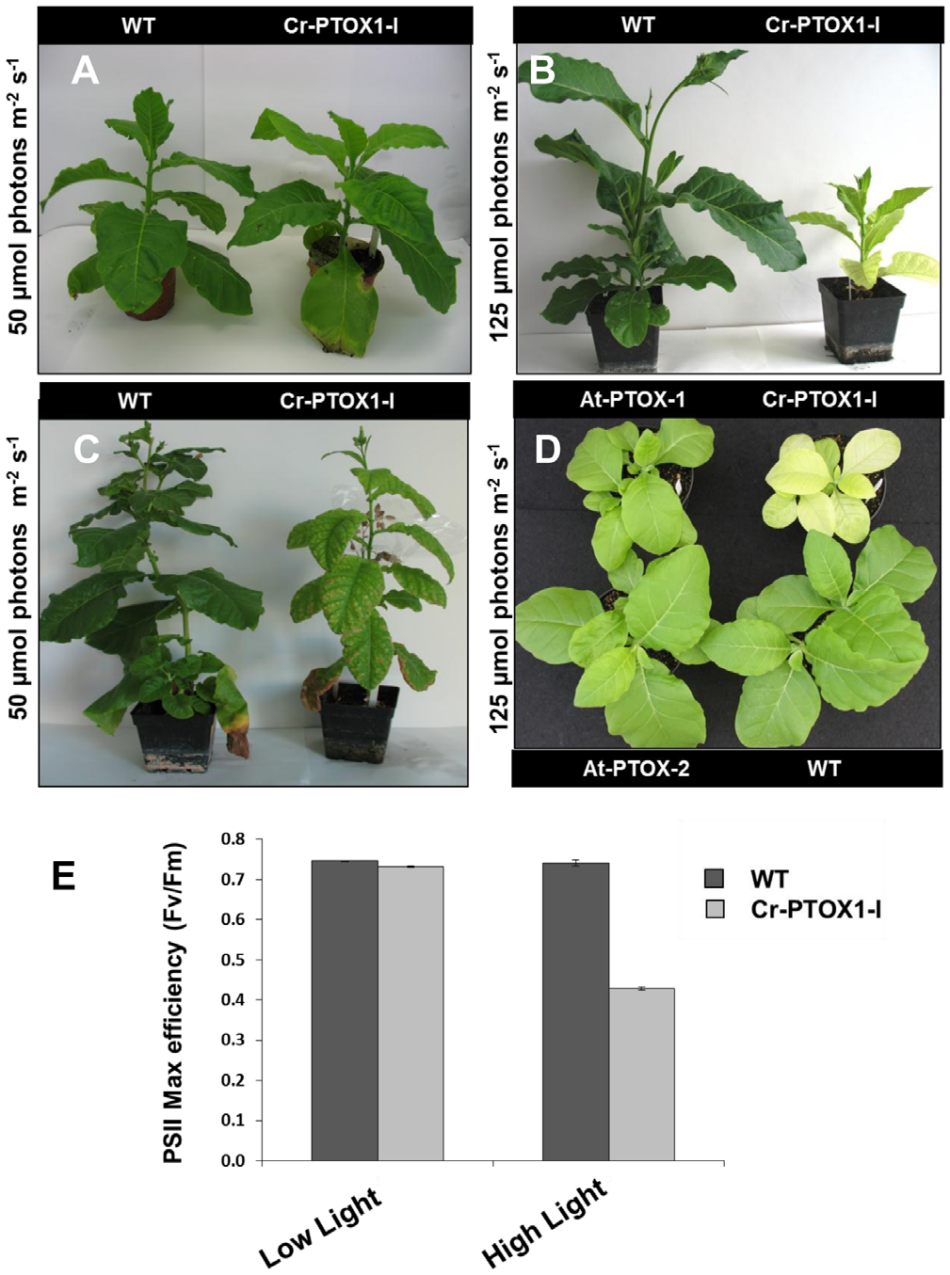
Impact of Cr-PTOX1 on plant under high light. Cr-PTOX1-I plants were grown at 50 µmol photons m^−2^ s^−1^ for a period of 4 weeks (A) and at 125 µmol photons m^−2^ s^−1^ for 10 weeks (B). (C) The plants grown in (B) were placed at 50 µmol photons m^−2^ s^−1^ for 6 weeks. The wildtype plants shown in (B) and (C) are shown for comparison purposes and were not germinated at the same time as the mutant. (D) Phenotypic comparison of two nuclear transformants, At-PTOX-1 and AT-PTOX-2, and transplastomic Cr-PTOX1-I plants at 4-week-old stage grown at 125 µmol photons m^−2^ s^−1^. (E) PSII maximum quantum efficiency was determined by recording Fv/Fm on attached 4–6 week-old healthy leaves of WT and Cr-PTOX1-I plants grown under low light (50 µmol photons m^−2^ s^−1^) and high light conditions (125 µmol photons m^−2^ s^−1^). The leaves were dark adapted for one hour before measuring fluorescence.

Cr-PTOX1-I plants grew normally under low light conditions (50 µmol photons m^−2^ s^−1^) (Figure 7A). However, Cr-PTOX1-I and all its progeny appeared very sensitive to higher light conditions (125 µmol photons m^−2^ s^−1^) and exhibited a quite distinct phenotype to that of WT, characterized by chlorotic leaves and significantly retarded growth (Figure 7B). However, the Cr-PTOX1-I line phenotype reverted back to a WT green colour when the light intensity was reduced from 125 µmol photons m^−2^ s^−1^ to 50 µmol photons m^−2^ s^−1^ (Figure 7C).

This yellowish and dwarfed phenotype had not been reported in earlier studies in which PTOX from *Arabidopsis thaliana* (At-PTOX) was expressed in tobacco via nuclear transformation [9], [30]. To check this directly, growth of the previously published nuclear transformants PTOX-1 and PTOX-2 [9] and the Cr-PTOX1-I transplastomic plant line were compared by growing them at an irradiance of 125 µmol photons m^−2^ s^−1^. The Cr-PTOX1-I transplastomic plants were clearly distinguishable from the nuclear transformants by their characteristic yellowish phenotype; both at early as well later stage of plant development (Figure 7D). No phenotypic difference was observed between the nuclear transformants (PTOX-1 and PTOX-2) and the wild type plants at this light intensity.

A major target for light damage is the PSII complex [28]. PSII activity in Cr-PTOX1-I expressed as the ratio of variable to maximum chlorophyll fluorescence (Fv/Fm) was 0.74 in low light (close to the value of 0.8 in WT [29]), but significantly lower (Fv/Fm = 0.43) in high-light grown transplastomic plants as compared to WT plants (Fv/Fm = 0.75), although it was restored to WT levels when returned to low light (Fv/Fm = 0.75) (Figure 7E). These data suggested that the Cr-PTOX1-I plant line was more susceptible to chronic photoinhibition than the WT.

## Discussion

In this study, the potential of tobacco chloroplasts to express and target foreign membrane proteins was tested. The results show that the PTOX1 membrane protein from *Chlamydomonas reinhardtii* was successfully expressed in tobacco chloroplasts and targeted to the thylakoid membrane network in an enzymatically active form. So far, there is only one report describing the expression of a foreign membrane protein via chloroplast transformation, the *Arabidopsis* inner envelope membrane protein, Tic40 [7]. However this work did not confirm whether chloroplast-expressed Tic40 was functional after its insertion into the chloroplast inner envelope.

Analysis by 2D-BN-PAGE suggested that Cr-PTOX1 is present in the membrane as a monomer and in a larger complex of approximately twice the mass (Figure 4C). Given the fact that mitochondrial alternative oxidase (AOX) exists in dimers [31] and that PTOX shares similar structural and functional characteristics with AOX [11], [32], it is reasonable to speculate that Cr-PTOX1 could form homodimers. Importantly BN-PAGE analysis did not provide evidence for association of Cr-PTOX1 with any of the major photosynthetic complexes of the thylakoid membrane such as Cytochrome b_6_f.

Immunoblot analysis using *C. reinhardtii* extracts as a comparison (Figure 3C) and the lack of a strongly stained band upon SDS-PAGE (Figure 3A) suggested that Cr-PTOX1 was not expressed at high levels in tobacco. Expression might be improved in future by modifying sequences important for translation. For example, the levels of 5-enolpyruvylshikimate-3-phosphate synthase (EPSPS) accumulation in tobacco plastids increased 10,000-fold range by simply using different 5′ UTRs from the same promoter [33] and a further >30 fold increase was observed when 14 amino acids from N-terminus of the green fluorescent protein (GFP) were fused to EPSPS [33]. We speculate that the high protein density of the thylakoid membrane and subsequent protein crowding might also be an important factor controlling expression.

The fluorescence assays conducted here to assess the activity of Cr-PTOX1 such as the post-illumination F_o_ rise (Figure 5), plastoquinol re-oxidation in the dark (Figure 6A) and the subsequent inhibition by propyl gallate (Figure 6C), a specific inhibitor of PTOX, confirmed that at least some of the expressed Cr-PTOX1 was present in an active conformation. The results of the chlorophyll fluorescence experiments closely mirrored previous studies showing that PTOX from *Arabidopsis* could be expressed in tobacco in an active form through transformation of the nucleus [9]. Likewise our data also provide evidence that transplastomic expression of enzymatically active Cr-PTOX1 was successful.

Electron flow through PTOX is thought to be a mechanism to prevent photoinhibition [15]. An intriguing finding of this study was the increased sensitivity, rather than resistance of the Cr-PTOX1-expressing plants to high light (Figure 7B). Under high light the Cr-PTOX1 expressing plant exhibited a dramatic phenotype, characterized by yellowish coloured leaves and significantly reduced growth (Figure 7B,D). A similar chlorotic phenotype has also been observed when plants with highly reduced levels of iron superoxide dismutase (FeSOD) levels are exposed to high light [34]. The FeSOD is a part of the ROS-scavenging system in plants [35] and therefore its disruption leads to the high accumulation of ROS, particularly superoxide (O_2_^−^), which by enhancing the rate of PSII damage made tobacco plants highly sensitive to photo-oxidative stress [34]. Expression of *Arabidopsis* PTOX (At-PTOX) in *E. coli* was also found to give rise to increased ROS-formation [30]. It is therefore possible that the enhanced production of ROS by Cr-PTOX1 under high light conditions could have rendered plants more sensitive to high light. However we found a very limited level of ROS damage to thylakoid membrane proteins in Cr-PTOX1-I using the Oxyblot™ kit (Millipore) (data not shown) and increased ROS production associated with the increased PTOX activity has yet to be shown for the nuclear transformants expressing At-PTOX [9].

The enhanced sensitivity of growth to high light was not reported in studies of the nuclear mutants over-expressing higher plant PTOX [9], [10], [30]. The growth difference between plastid and nuclear transformants (Figure 7D) could be due to a number of reasons: the level of expression of PTOX and consequently its activity might be much higher in the transplastomic plants than in the nuclear transformants, leading to increased ROS production with potential effects on cellular damage and signalling processes needed for high-light acclimation [36]; Cr-PTOX1 and At-PTOX might have intrinsically different enzymatic or regulatory properties; and the phenotype of the transplastomic plants might be the result of an indirect effect of expressing Cr-PTOX1 within the tobacco chloroplast such as inhibition of D1 synthesis during the PSII repair cycle [37].

In summary, the results of this study indicate that a foreign membrane protein PTOX1 from *C. reinhardtii* (Cr-PTOX1) was successfully expressed in an active form in tobacco chloroplasts. Our work highlights the potential of using chloroplasts as an expression platform for the production of membrane proteins, not just soluble proteins, although further work is needed to enhance the level of expression.

## Materials and Methods

### Plant material and growth conditions

*Nicotiana tabacum* cv Petit Havana was used in this study. Seeds were grown in magenta boxes on Murashige and Skoog (MS) medium [38] supplemented with 8 g L^−1^ agar and 30 g L^−1^ sucrose. Plants were grown on soil in a greenhouse as described previously [39]. Transplastomic plants sensitive to highlight were grown in a growth room at 25°C, 16 h light/8 h dark, photosynthetic photon flux of 50 µmol photons m^−2^ s^−1^ provided by cool white fluorescent bulbs and 30% of humidity. For high light, plants were grown at 25/20°C (day/night) in a controlled-environment of 16 h photoperiod, photosynthetic photon flux of 125 µmol photons m^−2^ s^−1^ provided from cool white fluorescent bulbs, and 40% humidity.

### Multiple sequence alignment and phylogenetic analysis

Phylogenetic analysis were performed using EBI's ClustalW2 (http://www.ebi.ac.uk/Tools/phylogeny/clustalw2_phylogeny/) [40]; trees were drawn either by neighbour-joining or Unweighted Pair Group Method with Arithmatic Mean (UPGMA) clustering method, which were viewed and drawn by Dendroscope Beta (version 3) (http://ab.inf.uni-tuebingen.de/software/dendroscope/) [41].

### Construction of chloroplast transformation vector expressing Cr-PTOX1

The cDNA sequence coding for PTOX1 of *Chlamydomonas reinhardtii* (GenBank accession number AF494290) was retrieved from GenBank and the chloroplast signal peptide was identified using ChloroP version 1.1 [20] and TargetP version 1.1 [21]. Codon usage of the sequences coding for the mature polypeptide was optimized according to the one preferred by the chloroplast machinery using codon usage table published by Kazusa DNA Research Institute (http://www.kazusa.or.jp/java/codon_table_java/). Using primer Cr-PTOX1-F-NdeI (GGCATACCATATGTATCCTTATGATGTTCCAGATTAT) and Cr-PTOX1-R-XbaI (GTTTATAATCTAGACTAGATA) Cr-PTOX1 coding sequence was amplified and cloned into the pHK40 vector [22] as an NdeI/XbaI fragment to generate plasmid pNA-PTOX1. The sequence TATCCTTATGATGTTCCAGATTAT encoding the influenza virus A hemaglutinin tag (HA-tag), YPYDVPDYA, was attached to the 5′-end of the *Cr-PTOX1* sequence for immunodetection from plant extracts.

### Transformation and regeneration of transplastomic plants

Transformation of the tobacco plastid genome and regeneration of transformed shoots was carried as described previously [39]. The primary shoots were analysed by PCR for the integration of transgenes into the tobacco plastome using a set of primers of which one primer, RPS-outside (TTCATGTTCCAATTGAACACTGTCCATT), sits on the plastid genome in a region outside the inserted sequences, and other primer was the corresponding forward primer, Cr-PTOX1-F-NdeI (GGCATACCATATGTATCCTTATGATGTTCCAGATTAT) used to amplify the gene for cloning. The positive shoots were subjected to two further rounds of regeneration under the same selection and then transferred to rooting medium. When sufficient root mass was obtained, these plants were transplanted to soil in green house to obtain seeds.

### Gel blot analysis

Transplastomic plants were tested for homoplastomy as described previously [39]. Briefly, total genomic DNA, extracted from 4–6 week-old leaves, was digested with BglII, electrophoresed in a 1% (w/v) agarose gel and transferred to a nylon membrane by overnight capillary transfer. A region of the plastome containing the site of integration (*rrn16* and *rps12/7*) was amplified by PCR from WT tobacco using primer RRN16-F (AATTCACCGCCGTATGGCTGACCGGCGA) and RPS12/7-R (GATCTTTCTCGATCAATCCCTTTGCCCCTCA), labelled with DIG High Prime DNA Labelling and Detection Starter Kit II (Roche Applied Science, Germany) and hybridised with digested genomic DNA from transplastomic plants. The specific signals were detected by exposing the membrane to X-ray film after incubating with anti-DIG (digoxigenin) antibodies and chloro-5-substituted adamantyl-1,2-dioxetane phosphate (CSPD) solution as described previously [42]. In the case of the RNA gel blot analysis, *Cr-PTOX1* was used as a probe.

### Protein extraction, SDS-PAGE and Western blot analysis

Total proteins from WT and Cr-PTOX1 expressing plants were extracted [43] and detection of Cr-PTOX1 in tobacco plants was carried out by SDS-PAGE [44] followed by immunoblotting using anti-HA tag (Roche Applied Science, Germany) or anti-PTOX1 antibodies (provided by Dr Xenie Johnson [16]). Thylakoids were extracted from tobacco leaves following the procedure as described in [45]. Differential extraction of thylakoid components as well as analysis of thylakoids by BN-PAGE and 2D-SDS-PAGE was carried as described previously [46]. Broad-range pre-stained multicolour molecular weight standards, Spectra™ (Fermentas, USA), were run alongside samples to determine the sizes of protein bands. The gels were stained with Coomassie Brilliant Blue ‘R-250’.

After electrophoresis, proteins were transferred to a nitrocellulose membrane using an iBlot® Dry Blotting System (Invitrogen, USA), incubated with primary antibodies overnight at 4°C, before being incubated with secondary antibodies conjugated with horseradish peroxidase (HRP). The secondary antibodies against rabbit IgG were detected using Enhanced Chemiluminescence (ECL) Detection kit (Amersham Pharmacia, UK) on an X-ray film (Kodak, USA).

### Differential extraction of membrane proteins

Membranes containing approximately 20 µg of chlorophyll were divided into three aliquots and centrifuged at 13,000 rpm at 4°C for 20 min in an accuSpin® Micro Centrifuge (Fischer Scientific, UK). Subsequently, the pellets were resuspended in 100 µl of various buffers: PEB alone (50 mM HEPES/KOH pH 7.5, 1 mM EDTA, 2 mM DTT, 10 mM potassium acetate, 5 mM magnesium acetate, protease complete inhibitor cocktail (Roche Applied Sciences, Germany) 1 mini tablet added to each 5 ml of buffer) [47], one aliquot in PEB with 2 M NaCl, pH 8.0 and one aliquot in 20 mM CAPS, pH 12.0. The samples were then treated two times with freeze thaw cycle 30 min at −80°C and 15 min at room temperature and pelleted by centrifuging at 100,000 g for 30 min at 4°C in an ultracentrifuge (Ultracentrifuge T-100; Beckman Coulter, UK; Rotor model: TLA 120.1). The supernatant was removed carefully. The pellet fraction, as well as supernatant, were solubilised in SDS solubilisation buffer for 45 min at room temperature and then loaded onto an SDS-PAGE (1 µg of chlorophyll per well for membranes and equivalent fraction of total for supernatant) for analysis.

### Chlorophyll fluorescence measurements

Chlorophyll fluorescence measurements were performed out using pulse-modulated fluorometer (DUAL PAM 2000, Walz, Germany) equipped with DUAL-PAM-100 measuring system (http://www.walz.com/products/chl_p700/dual-pam-100/introduction.html). The chlorophyll fluorescence was recorded by DUAL-PAM v1.11 software [48]. All the measurements were carried out at room temperature. The minimal fluorescence level (F_o_) and the maximal fluorescence levels (F_m_) were taken using a saturation pulse of 6,000 µmol photons m^−2^ s^−1^ for 0.6 sec in dark-adapted leaves. The plants were dark-adapted for at least 30 min before the measurements were made.

### Inhibitory treatment of leaf discs by propyl gallate

The propyl gallate treatment was performed as described in [9]. Briefly leaves from 8-week-old plants were stripped of lower epidermis and cut into small discs. These discs were soaked in 0.5% (v/v) methanol/water with or without 1 mM propyl gallate for three hours in the dark.
